# Spatial Patterns of Gene Expression in Bacterial Genomes

**DOI:** 10.1007/s00239-020-09951-3

**Published:** 2020-06-06

**Authors:** Daniella F. Lato, G. Brian Golding

**Affiliations:** Department of Biology, McMaster Univeristy, 1280 Main St. West, Hamilton, ON L8S 4K1 Canada

**Keywords:** Genome location, Gene expression, Origin of replication, Terminus of replication, Bacteria, Genomics

## Abstract

Gene expression in bacteria is a remarkably controlled and intricate process impacted by many factors. One such factor is the genomic position of a gene within a bacterial genome. Genes located near the origin of replication generally have a higher expression level, increased dosage, and are often more conserved than genes located farther from the origin of replication. The majority of the studies involved with these findings have only noted this phenomenon in a single gene or cluster of genes that was re-located to pre-determined positions within a bacterial genome. In this work, we look at the overall expression levels from eleven bacterial data sets from *Escherichia coli*, *Bacillus subtilis*, *Streptomyces*, and *Sinorhizobium meliloti*. We have confirmed that gene expression tends to decrease when moving away from the origin of replication in majority of the replicons analysed in this study. This study sheds light on the impact of genomic location on molecular trends such as gene expression and highlights the importance of accounting for spatial trends in bacterial molecular analysis.

**Electronic Supplementary Material:**

The online version of this article (10.1007/s00239-020-09951-3) contains supplementary material, which is available to authorized users.

## Introduction

Gene expression in bacteria is complex and highly controlled. The regulation of bacterial gene expression is a crucial component of bacterial survival in order for these organisms to modulate gene expression and alter phenotypic properties such as growth rate (Garmendia et al. 2018) and motility (Ravichandar et al. 2017). Gene expression can be controlled through a variety of promoters, physical chromosome structure, and the DNA replication machinery. Therefore, different genes can be under distinct methods of regulation and be expressed at fluctuating levels depending on environmental conditions or growth stage. This variation in expression can be influenced by a myriad of effects such as differences in codon bias (Gutman and Hatfield 1989; Sharp et al. 1989; Buchan et al. 2006; Cannarozzi et al. 2010; Quax et al. 2015), gene orientation (Zeigler and Dean 1990; Kunst et al. 1997; Price et al. 2005), replication (Rocha 2004b; Washburn and Gottesman 2011; Block et al. 2012; Garmendia et al. 2018), and chromosomal location (Sharp et al. 2005; Couturier and Rocha 2006; Morrow and Cooper 2012). These phenomena can create predictable patterns that can be observed in many molecular traits across many bacterial species.

One set of patterns is related to the physical location of genes on the chromosome. Some studies have found certain genes and groups of genes to be expressed periodically around the chromosome. Wright et al. (2007), looked at statistically correlated gene pairs in *E. coli* and found that they are often separated by 100 kilobase pairs (Kbp) and are often located in areas of high transcription. Other studies of *E. coli* observed that sections of the chromosome with increased transcription rates were periodically found throughout the genome over 700–800Kbp ranges (Jeong et al. 2004). It is speculated that this periodic phenomenon is due to a combination of physical constraints of the chromosome, such as histones and supercoiling, and DNA composition (Jeong et al. 2004; Képes 2004; Peter et al. 2004; Allen et al. 2006; Block et al. 2012). Prior research on spatial molecular trends when moving from the origin of replication to the terminus has determined that gene expression (Sharp et al. 2005; Couturier and Rocha 2006; Morrow and Cooper 2012) and gene dosage (Cooper and Helmstetter 1968; Schmid and Roth 1987; Rocha 2004a; Block et al. 2012; Sauer et al. 2016) are increased near the origin, and genes become less conserved with increasing distance from the origin (Couturier and Rocha 2006). Additionally, substitution rates (non-synonymous (d*N*), synonymous (d*S*)), and the d*N*/d*S* ratio, increase with distance from the origin of replication (Cooper et al. 2010; Morrow and Cooper 2012). The variation in molecular trends with genomic location has been suspected to be due to a number of complicated and intertwining factors such as transposon insertion events (Gerdes et al. 2003), gene order and conservation (Mackiewicz et al. 2001; Flynn et al. 2010), replication (Couturier and Rocha 2006), and nucleotide composition (Mackiewicz et al. 1999; Karlin 2001; Sharp et al. 2005).

Gene expression in particular consistently varies with distance from the origin of replication. A number of previous studies have analysed this spatial trend in a variety of bacteria such as *E. coli*, *Brucella*, and *Vibrio*. Both large- (Sharp et al. 2005; Couturier and Rocha 2006) and small-scale studies (Schmid and Roth 1987; Morrow and Cooper 2012; Block et al. 2012; Bryant et al. 2014; Garmendia et al. 2018) have detected decreasing gene expression values as genomic distance increases away from the origin of replication. However, the majority of these studies often only look at a single gene or cluster of genes and promoters (Schmid and Roth 1987; Block et al. 2012; Bryant et al. 2014; Garmendia et al. 2018). In these studies, genes or gene clusters are experimentally moved to pre-determined locations around the replicon. This type of experiment can lead to biases stemming from the original location of the genes and the relative distance from the origin of replication. Additionally, the genes chosen are often selected because of their ability to be easily moved to various genomic locations. Choosing specific genes to manipulate and move around bacterial genomes is fundamental to understand how the location of a gene on a chromosome impacts its expression. However, observing one gene does not provide us with a complete picture of what is happening with gene expression from a genomic viewpoint.

Although many studies have found that gene expression decreases with increasing distance from the origin of replication, it is unclear if this phenomenon is persistent across diverse genomes and bacterial species. In this work, we aim to answer this question by looking at the overall expression levels of all genes within eleven gene expression data sets from bacterial genomes of *Escherichia coli*, *Bacillus subtilis*, *Streptomyces*, and *Sinorhizobium meliloti*. These bacteria inhabit a variety of different environments and cover a range of genomic structures and replication strategies. Some of the bacteria in this study have a single circular (*E. coli* and *B. subtilis*) or linear chromosome (*Streptomyces*) containing its genome, while others have the genome split up into multiple replicons (*S. meliloti*). Each of these genomic structures requires precise coordination between transcription and translation in order to replicate efficiently. This selection of bacterial taxa provides a sample that covers broad lifestyles as well as representing a number of divergent phylogentic lineages, providing a diverse sample for answering if gene expression decreased with increasing distance from the origin of replication in across diverse bacterial genomes and species. Using whole genome expression data obtained from the GEO database (Barrett et al. 2012), we are able to observe genomic expression patterns in natural populations devoid of stress, while accounting for bidirectional replication. We have confirmed that gene expression indeed tends to be higher near the origin of replication and decreases with increasing distance from the origin. Understanding how the distance of a gene from the origin of replication can impact the expression level assists in explaining other spatial distance trends such as gene essentiality, gene conservation, and mutation rates.

## Materials and Methods

### Expression Data

The bacteria chosen for this analysis were *E. coli*, *B. subtilis*, *Streptomyces*, and *S. meliloti*. These bacteria inhabit a variety of different living environments and have contrasting genomic structures (i.e. circular, linear, multi-repliconic), providing a well-rounded sample for this analysis. Although *E. coli*, *B. subtilis*, and *Streptomyces* contain small plasmids, they are not considered multi-repliconic bacteria, and therefore, their plasmids were not included in this analysis. *S. meliloti* is a multi-repliconic bacteria and its two large secondary replicons were included in the analysis (pSymA and pSymB). The replicons of *S. meliloti* are known to differ in genetic content, and therefore, all analyses were performed on each individual replicon of *S. meliloti*.

Gene expression data for *E. coli*, *B. subtilis*, *Streptomyces*, and *S. meliloti* were downloaded from the Gene Expression Omnibus (GEO) (Barrett et al. 2012). The expression data sets for this analysis were only RNA-seq data sets for control data, where this was defined as the bacteria being grown in environments absent of any stress. Using strictly raw RNA-seq expression data allows the normalization to be standardized across all data sets, making the data sets directly comparable. The additional condition of using expression data where the bacteria were grown in control- or stress-free environments again allows for direct comparisons to be made between spatial gene expression trends between these bacterial species. Due to these constraints on our data, we were only able to retrieve a total of 11 gene expression data sets from GEO for this analysis.

Pseudogenes were excluded from this analysis. A complete list of expression data used is found in Supplementary Table S1. Correlation of gene expression across data sets was assessed for each bacteria with multiple data sets. For a detailed protocol, see Supplementary files on GitHub at https://github.com/dlato/Spatial_Patterns_of_Gene_Expression.git.

### Normalization

The raw counts from control populations for each data set were used and normalized using the TMM method (Robinson and Oshlack 2010). Raw counts were normalized to Counts Per Million (CPM) in R using the edgeR package (Robinson et al. 2010). After normalization, any data sets that had multiple replicates were combined by finding the median CPM between replicates for each annotated gene. Only genes that had expression values in all data sets were used for this analysis.

### Genomic Position

To relate the median CPM gene expression values to position in the genome, a custom Python script was written to determine the midpoint position of each annotated gene in the bacterial genome. This allowed a single position location for each gene, which simplifies the following regression calculations.

### Origin and Bidirectional Replication

**Fig. 1:**
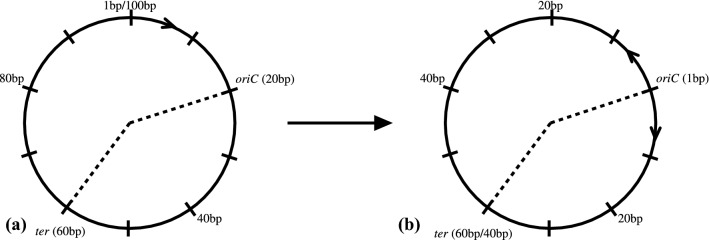
Schematic of the transformation used to scale the positions in the genome to the origin of replication and account for bidirectional replication. Circle (**a**) represents the original replicon genome without any transformation. Circle (**b**) represents the same replicon genome after the transformation. The origin of replication is denoted by “*oriC*” and the terminus of replication is denoted by “*ter*”. The dashed line represents the two halves of the replicon separated by replication. The replicon genome in this example is 100 base pairs in length. Every 10 base pairs are denoted by a tick on the genome. The origin in (**a**) is at position 20 in the genome and is transformed in (**b**) to become position 1. The terminus is at position 60 in (**a**) and position 60 and 40 in (**b**). The terminus has two positions in (**b**) depending on which replicon half is being accounted for. If the replication half to the right of the origin is considered, the terminus will be at position 40. If the replication half to the left of the origin is considered, the terminus will be at position 60. Position 40 in (**a**) becomes position 20 in (**b**). Position 80 in (**a**) becomes position 40 in (**b**), because of the bidirectional nature of bacterial replication. “bp” denotes base pairs

For each bacteria in this analysis, the beginning of the origin of replication was denoted as the beginning of the *oriC* region for the chromosomal replicons, and the beginning of the *repC* (Pinto et al. 2011) region for the secondary replicons of *S. meliloti* (Supplementary Table S2). This origin of replication position was calibrated to be the beginning of the genome, or position 1, and remaining positions in the genome were all scaled around this origin of replication (Fig. 1).

To determine if specifying a single nucleotide as the origin of replication would alter the results, we performed permutation tests. These tests shuffled the *oriC* position by 10,000 base pairs (bp) increments in each direction from the original origin (data not shown) to a maximum of 100,000bp in each direction. These results showed that moving the origin of replication does not affect the results of the analysis (data not shown).

The terminus of replication was determined using the Database of Bacterial Replication Terminus (DBRT) (Kono et al. 2011). DBRT uses the prediction of *dif* sequences as a proxy for the terminus location because the *dif* sequences are located in the replication termination region of the chromosome (Clerget 1991; Blakely et al. 1993). For pSymA and pSymB of *S. meliloti*, the terminus is not listed in the database; thus, the terminus location was assigned to the midpoint between the origin of replication and the end of the replicon. Replication in the linear chromosome of *Streptomyces* begins at the origin of replication, located to the right of the middle of the replicon (Heidelberg et al. 2000), and terminates at each end of the chromosome arms (Heidelberg et al. 2000) (Supplementary Table S2).

The origin scaling and bidirectional replication transformations were done in R (R Development Core Team 2014) and inferences about gene expression were made while recording their distance from the origin of replication. A diagram of this transformation is outlined in Figure 1.

**Fig. 2:**
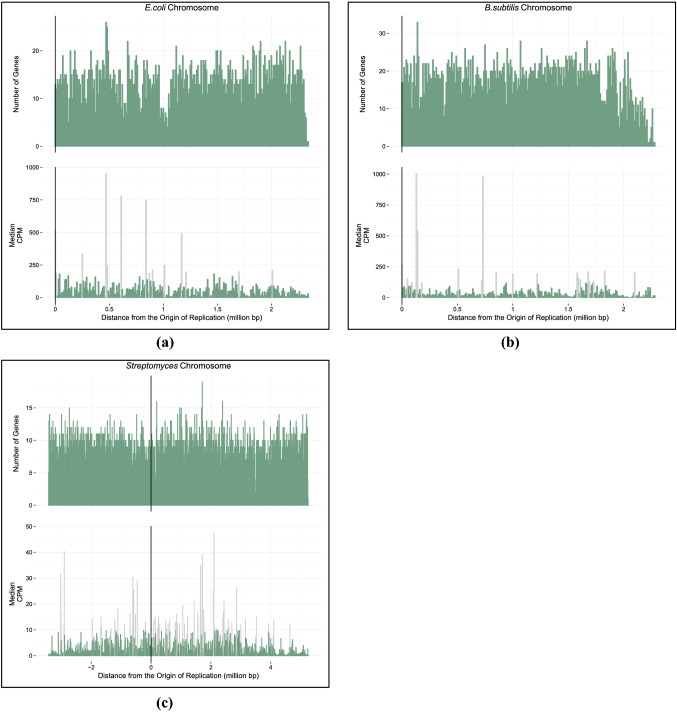
The top bar graphs show a count of the total number of genes (*y*-axis) at each position (*x*-axis) in the genome of *E. coli* (**a**), *B. subtilis* (**b**), and *Streptomyces* (**c**). The bottom bar graphs show the median expression data along the genomes of *E. coli* (**a**), *B. subtilis* (**b**), and *Streptomyces* (**c**). The origin of replication is indicated by a black vertical line. For *E. coli* and *B. subtilis*, the distance from the origin of replication is on the *x*-axis beginning with the origin of replication denoted by position zero on the left and the terminus indicated on the far right. For *Streptomyces,* the origin of replication is denoted by position zero. The genome located on the shorter chromosome arm (to the left of the origin) has been given negative values, while the genome on the longer chromosome arm (to the right of the origin) has been given positive values. The *y*-axis of the bottom graph indicates the total median CPM expression values found at each position of the *E. coli* (**a**), *B. subtilis* (**b**), and *Streptomyces* (**c**) genomes. Each bar represents a section of the genome that spans 10,000 base pairs. Light coloured bars represent statistical outliers

**Fig. 3:**
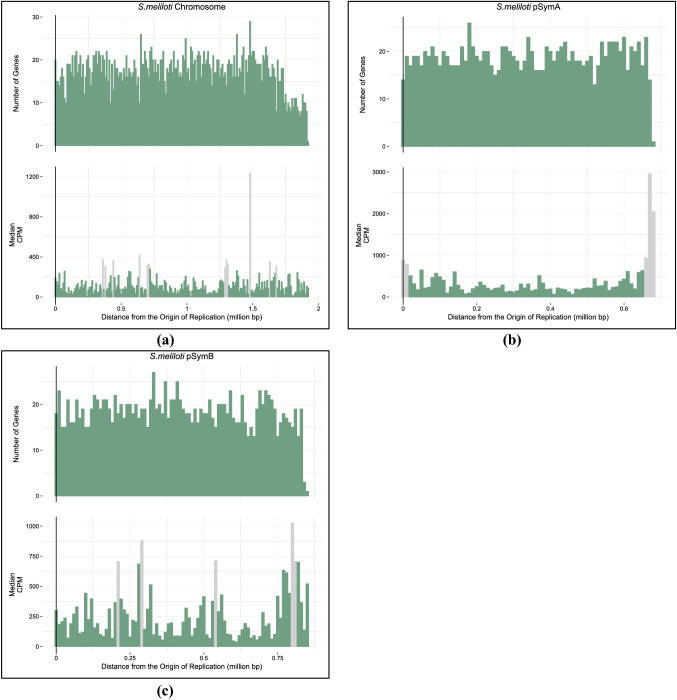
The top bar graphs show a count of the total number of genes (*y*-axis) at each position (*x*-axis) of the replicons of *S. meliloti*: chromosome (**a**), pSymA (**b**), and pSymB (**c**). The bottom bar graphs show the median expression data along the *S. meliloti* replicons: chromosome (**a**), pSymA (**b**), and pSymB (**c**). The origin of replication is indicated by a black vertical line. The distance from the origin of replication is on the *x*-axis beginning with the origin of replication denoted by position zero on the left and the terminus indicated on the far right. The *y*-axis of the bottom graph indicates the total median CPM expression values found at each position of the *S. meliloti* replicons: chromosome (**a**), pSymA (**b**), and pSymB (**c**). Each bar represents a section of the genome that spans 10,000 base pairs. Light coloured bars represent statistical outliers

*E. coli*, *B. subtilis*, and all replicons of *S. meliloti* have a terminus of replication which is located roughly equidistant from the origin of replication (Supplementary Table S2). These bacteria, therefore, have approximately symmetrical chromosomal arms and as a result have genomic position labelling in Figures 2 and 3, accounting for bidirectional replication. *Streptomyces*, on the other hand, is an acrocentric linear chromosome with one chromosomal arm being much shorter than the other (see Figure 2). The genomic position labelling of *Streptomyces* in Figure 2 has negative numbers to indicate the shorter chromosome arm and positive numbers indicating the longer chromosome arm.

### Average Gene Expression

The average gene expression per genome was calculated for each bacterial replicon. This was computed by taking the arithmetic mean of all normalized CPM gene expression values for the entire replicon.

A single median CPM per 10 Kbp section of each bacterial genome was calculated. The gene expression information was summarized in bar graphs in R using ggplot2 (Wickham 2009) (Figures 2 and 3). Supplementary interactive figures can be found on GitHub (https://github.com/dlato/Spatial_Patterns_of_Gene_Expression.git).

### Linear Regression

To assess the statistical significance of changes in expression with genomic position, a simple linear regression was performed in R (R Development Core Team 2014). An average CPM expression value was calculated for each 10 Kbp region of the genome. This was calculated by taking the sum of all CPM expression values over a 10 Kbp region of the genome and dividing this by the total number of genes present in that 10 Kbp segment. A linear regression was performed on these 10 Kbp average expression values to determine if there was a significant correlation between gene expression and the distance from the origin of replication. Statistical outliers in this data set were removed from the linear regression. Outliers were defined as being outside the first quartile minus 1.5 times the interquartile range and the third quartile plus 1.5 times the interquartile range. Additional linear regressions on a per gene basis, non-average expression values, and total additive expression values were also calculated. These results and methods can be found in the Supplementary Material (Supplementary Tables S3- S5).

The total number of protein coding genes was determined for each 10 Kbp region of the genome. To assess the statistical significance of the total number of genes in each 10 Kbp region of the genome and position in the genome, a simple linear regression was performed in R (R Development Core Team 2014).

A supplementary test to determine if gene expression differs between the leading and lagging strands of each bacterial replicon was performed. A two-sample Wilcox test was computed in R (R Development Core Team 2014) to compare expression of genes on the leading strand and the lagging strand. We found that there was no significant difference between gene expression on the leading and lagging strand in most of the bacterial replicons. The exceptions to this were *Streptomyces* and the chromosome of *S. meliloti*, which had a significant difference between gene expression on the leading and lagging strand, with higher gene expression on the leading strand. Full results can be found in the Supplementary Material. The percent of genes that reside on the leading strand of the various bacterial replicons was between approximately 54% and 74% (see Supplementary Material).

## Results and Discussion

### Origin and Bidirectional Replication

Bacterial chromosome replication begins at the origin of replication and proceeds away from the origin in both directions (Prescott and Kuempel 1972). Bidirectional replication affects the genomic location of the farthest point from the origin. Replication concludes at the terminus (Prescott and Kuempel 1972) which in circular replicons is usually located opposite from the origin (Kono et al. 2011). However, in some bacteria the terminus is not exactly opposite from the origin. In a case like this, some of the distance measurements will only account for one of the replication halves (Fig. 1). However, due to the nearly symmetrical location of the terminus to the origin, this effect is small.

In this analysis, a single base was chosen to represent the origin of replication. In reality, the origin of replication is often a number of base pairs long and choosing the first nucleotide position of this *oriC* region or the last nucleotide of this region may alter the subsequent bidirectional replication transformations and results. We performed permutation tests (data not shown) to determine the impact of altering the location of the origin of replication position. These results from our origin of replication permutation tests determined that moving the origin of replication does not affect the overall trends, providing a robust check for origin of replication location.

**Table 1:** Arithmetic mean gene expression calculated across all genes in each replicon

Bacteria and replicon	Average expression value (CPM)
*E. coli* chromosome	176.009
*B. subtilis* chromosome	186.533
*Streptomyces* chromosome	6.453
*S. meliloti* chromosome	286.723
*S. meliloti* pSymA	764.793
*S. meliloti* pSymB	628.318

Expression values are represented in Counts Per Million

**Table 2:** Linear regression results of average expression and distance from the origin of replication

Bacteria and Replicon	Regression slope of the change in gene expression with distance from the origin of replication
***E. coli* Chromosome**	**− 3.65 × 10**^−5^***
***B. subtilis*Chromosome**	**− 2.48 × 10**^−5^**
***Streptomyces*Chromosome**	**− 1.41 × 10**^−7^**
*S. meliloti* Chromosome	NS
*S. meliloti* pSymA	NS
*S. meliloti* pSymB	NS

The average expression values were calculated by dividing the total counts per million expression value per 10kb section of the genome by the total number of genes in the respective 10kb section. Linear regression was calculated after the origin of replication was moved to the beginning of the genome and all subsequent positions were scaled around the origin accounting for bidirectional replication. Statistical outliers were removed from this linear regression calculation. All results are marked with significance codes as followed: < 0.001 = ‘***’, 0.001 < 0.01 = ‘**’, 0.01 < 0.05 = ‘*’, > 0.05 = ‘NS’. Bold indicates a significant negative trend

**Table 3:** Linear regression analysis of the total number of protein coding genes per 10 Kbp along the genome of the respective bacteria replicons

Bacteria and Replicon	Regression slope of the change in number of genes with distance from the origin of replication
*E. coli* Chromosome	NS
*B. subtilis* Chromosome	− 3.00 × 10^−6^***
*Streptomyces* Chromosome	NS
*S. meliloti* Chromosome	− 1.99 × 10^−6^***
*S. meliloti* pSymA	NS
*S. meliloti* pSymB	− 4.11 × 10^−6^*

Linear regression was calculated after the origin of replication was moved to the beginning of the genome and all subsequent positions were scaled around the origin accounting for bidirectional replication. All results are marked with significance codes as followed: < 0.001 = ‘***’, 0.001 < 0.01 = ‘**’, 0.01 < 0.05 = ‘*’, > 0.05 = ‘NS’

### Average Gene Expression

A summary of the average gene expression values per bacterial replicon can be found in Table 1. Most of the bacterial replicons have an average normalized expression value between 175 CPM - 765 CPM (Table 1). *Streptomyces* has an average gene expression value that is about two orders of magnitude lower than the other bacterial replicons (Table 1). This could be because there was only one data set available for this analysis (see Supplementary Table S1), and the mapped reads were assigned using the Galaxy streCoel (*Streptomyces coelicolor* 07/01/1996) Assembly (Afgan et al. 2018). This particular assembly and workflow may be why the *Streptomyces* gene expression data has consistently lower normalized CPM values across the genome compared to the other bacterial replicons which use a different suite of software including the Tuxedo Protocol (Trapnell et al. 2012).

### Linear Regression

The average CPM gene expression values were calculated over 10 Kbp regions. A linear regression was performed on those values to determine if there was a significant trend correlating gene expression and distance from the origin of replication. Gene expression decreases when moving away from the origin of replication for the chromosomes of *E. coli*, *B. subtilis*, and *Streptomyces* (Table 2). We were unable to detect a significant linear regression coefficient estimate for all replicons of *S. meliloti*. Previous work in similar bacterial species looking at the distribution of highly expressed (Couturier and Rocha 2006) and orthologous genes (Morrow and Cooper 2012) also found genes with higher expression values to be concentrated near the origin of replication. Our results are consistent with these studies as we see a decrease in gene expression with increasing distance from the origin of replication. All linear regression and supporting statistical information for the gene expression trends are found in Table 2. We performed additional statistical tests to look at how using different averaging methods for the gene expression values potentially altered the regression results. Some of these averaging methods included average gene expression over 10 Kbp regions of the genome, and the total added expression over 10 Kbp genomic regions. A full list of supplementary tests can be found in the Supplemental Material. We looked at the relationship between these averaged values and distance from the origin of replication and showed that there was no difference in averaging methods, and we still see gene expression decrease with increasing distance from the origin of replication. See Supplementary material for detailed methods of the additional regression tests.

Having higher gene expression values near the origin of replication has been linked to physical constraints and processes of the bacterial replicon (Képes 2004; Peter et al. 2004; Jeong et al. 2004; Allen et al. 2006; Block et al. 2012). For example, replication errors are thought to increase as replication moves farther from the origin of replication (Courcelle 2009). This impacts the placement of highly expressed and important genes where errors in replication could be detrimental to the gene product and the organism. Therefore, genes that are highly expressed and also essential to the survival of the organism might often be located near the origin of replication and on the leading strand to further avoid collisions between DNA and RNA polymerase (Rocha 2004b; Washburn and Gottesman 2011; Block et al. 2012). Genes that are part of the core genome of bacteria are typically located near the origin of replication (Sharp et al. 2005; Couturier and Rocha 2006; Flynn et al. 2010). These core genes make up the majority of bacterial genomes, so intuitively, we should have a higher concentration of genes near the origin of replication. We determined that the total number of protein coding genes per 10 Kbp decreases with distance from the origin of replication (Table 3). A higher concentration of genes is near the beginning of the genome, where we see increased expression, and a lower concentration of genes is near the terminus, where we observed decreased expression.

A number of studies suggest that it is the essentiality or function of the gene that impacts gene expression and organization of genes on the chromosome (Rocha and Danchin 2003; Rocha 2008). In particular, Couturier and Rocha (2006) found that only genes associated with transcription/translation were located close to the origin of replication, while other highly expressed genes are distributed randomly with respect to genomic location. To address this finding, we utilized the functional data available on the Clusters of Orthologous Groups of proteins (COG) database to assess how the functionality of genes change with distance from the origin of replication. A full account of the methods is found in the Supplementary Material. We found no clear pattern of genes with any functional COG category consistently being located near the origin of replication. This included genes that are associated with transcription and translation, which did not have a consistent correlation with distance from the origin of replication across all bacteria in this analysis. A full list of significant linear regression coefficients for all 24 COG functional categories can be found in the Supplementary Material. The lack of clear trends in functional categories changing with distance from the origin of replication leads us to believe that there may be mechanisms other than gene function dictating genomic gene expression trends in bacterial genomes.

Gene dosage appears to play an important role in the location of genes along bacterial replicons (Cooper and Helmstetter 1968; Schmid and Roth 1987; Rocha 2004a; Couturier and Rocha 2006; Block et al. 2012; Sauer et al. 2016). When gene expression is saturated, gene dosage can be used to alter transcription (Couturier and Rocha 2006). This has implications for rapid growth periods in bacteria, allowing tighter control of growth in varying environmental conditions (Couturier and Rocha 2006). Faster growing species require overlapping replication cycles to allow replication to keep up with growth (Helmstetter 1996). This should therefore correlation with the strength in gradients of expression with distance from the origin of replication (Morrow and Cooper 2012). This allows for increased expression for genes replicated earlier, and decreased expression for genes replicated later (Sharp et al. 1989; Mira and Ochman 2002; Couturier and Rocha 2006; Dryselius et al. 2008) Both gene dosage and the growth rate of a bacteria could provide a mechanism by which selection could act to influence the locations of genes along bacterial replicons. The high concentration of highly expressed genes located near the origin of replication could be influenced by additional selective forces such as translational efficiency which can alter codon usage bias (Ikemura 1985; Kanaya et al. 1999; Sharp et al. 2005; Morrow and Cooper 2012).

We did not detect a significant relationship between gene expression and distance from the origin of replication for the replicons of *S. meliloti* (chromosome, pSymA and pSymB). Gene expression in this bacteria is not as well studied as the other bacteria used in this analysis (Martens et al. 2008). In our search for expression data, we identified fewer appropriate studies for *S. meliloti* to include in our data analysis. A smaller amount of gene expression data may be biasing the non-significant correlation between gene expression and distance from the origin of replication in this *S. meliloti*.

It has been suggested that the leading strand is favoured for the location of highly expressed genes to allow faster DNA replication and lower transcriptional losses (Brewer 1988). We found no statistical evidence for the leading strand to have higher expression levels compared to the lagging strand in most of the bacterial replicons and have concluded that this is likely not driving the results of decreased gene expression with increased distance from the origin of replication. Previous studies have determined that the main factor that influences if a gene is on the leading or lagging strand is the essentiality of that particular gene, not expression (Rocha and Danchin 2003; Zheng et al. 2015). The number of bacterial genes on the leading strand varies between approximately 45 to 90% (Rocha 2002; Zivanovic et al. 2002; Koonin 2009; Mao et al. 2012). The bacterial replicons used in this analysis fall with this range, and therefore, the leading and lagging strands are not influencing the results (see Supplementary Material).

Areas of the bacterial genomes with extremely high gene expression (Supplementary Table S6) are regions that encode proteins involved in processes such as DNA repair and replication, RNA synthesis, metabolism, and ribosomal proteins. We expect these regions to have much higher expression levels compared to the rest of the genome because they encode proteins that are crucial to translation and replication processes. Shockingly, when accounting for bidirectional replication we see that some riboproteins in *E. coli*, *B. subtilis*, and *S. meliloti* are not always located close to the origin of replication and can be located up to 1.49 million base pairs (Mbp) away from the origin of replication (in the case of the chromosome of *S. meliloti*, see Supplementary Table S6 for more details).

## Conclusions

The genomic location of a bacterial gene has a profound impact on the expression levels of that gene. Previous studies have focused on a small subset of genes (Schmid and Roth 1987; Block et al. 2012; Bryant et al. 2014; Garmendia et al. 2018) or expression trends in one bacterial species (Schmid and Roth 1987; Block et al. 2012; Morrow and Cooper 2012; Bryant et al. 2014; Garmendia et al. 2018). Here, we assess gene expression levels across all protein coding genes within the bacterial genomes of *E. coli*, *B. subtilis*, and *Streptomyces* and show that there is a relationship with distance from the origin of replication. Most replicons in this study show that genes that are closer to the origin of replication have a higher expression level when compared to genes that are located farther from the origin of replication. This spatial variation is not unique to gene expression; other molecular trends such as gene conservation (Couturier and Rocha 2006) and substitution rate (Cooper et al. 2010; Morrow and Cooper 2012) also vary with distance from the origin. It is important to realize that the location of a gene within the genome will impact various molecular trends of that segment of DNA and may assist in explaining other phenomenon related to that gene. Further analysis on the spatial trends of other molecular traits such as substitution rate and gene essentiality will create a base of information on what molecular trends genomic location can alter.

## Electronic Supplementary Material

Below is the link to the electronic supplementary material.

Electronic supplementary material 1 (PDF 657 kb)
