# Supplementary material for: Spatial Patterns of Gene Expression in Bacterial Genomes
Source: J Mol Evol. 2020 Jun 6;88(6):510–20. doi: 10.1007/s00239-020-09951-3 (PMC7324424; doi:10.1007/s00239-020-09951-3)
Supplement: Supplementary file 1 — Electronic supplementary material 1 (PDF 657 kb) [file 239_2020_9951_MOESM1_ESM.pdf]

Title: Spatial Patterns of Gene Expression in Bacterial Genomes

Authors: Daniella F Lato and G Brian Golding

Journal: Journal of Molecular Evolution

Corresponding Author Information:
G. Brian Golding
McMaster Univeristy
Department of Biology
1280 Main St. West
Hamilton, ON
Canada
L8S 4K1
Email: golding@mcmaster.ca

Supplementary Material

All supplemental information including interactive graphs of the expression data, and pdf versions of all figures from the paper and supplement can be found on `GitHub` at `https://github.com/dlato/Spatial_Patterns_of_Gene_Expression.git`.

Interactive Graphs

The normalized gene expression data is available as a interactive graph for each bacterial replicon. The user can use their mouse to hover over gene expression points to determine the National Centre for Biotechnology Information (NCBI) gene Id. This Id can be searched in the NCBI website (`https://www.ncbi.nlm.nih.gov/`) to obtain more information on that particular gene. These interactive graphs are listed on `GitHub` as files:

- *E. coli*: “ecoli_gene_exp_interactive_graph.html”
- *B. subtilis*: “bsubtilis_gene_exp_interactive_graph.html”
- *Streptomyces*: “streptomyces_gene_exp_interactive_graph.html”
- *S. meliloti* Chromosome : “smeliloti_chromosome_gene_exp_interactive_graph.html”
- *S. meliloti* pSymA: “smeliloti_pSymA_gene_exp_interactive_graph.html”
- *S. meliloti* pSymB “smeliloti_pSymB_gene_exp_interactive_graph.html”

Gene Expression Data

Bacteria Strain/Species	GEO Accession Number	Date Accessed	NCBI Accession Genome Used For Gene Position
E. coli K12 MG1655	GSE60522	December 20, 2017	U00096
E. coli K12 MG1655	GSE73673	December 19, 2017	
E. coli K12 MG1655	GSE85914	December 19, 2017	
E. coli K12 DH10B	GSE98890	December 19, 2017	
B. subtilis 168	GSE104816	December 14, 2017	NC_000964
B. subtilis 168	GSE67058	December 16, 2017	
B. subtilis 168	GSE93894	December 15, 2017	
S. coelicolor A3	GSE57268	March 16, 2018	AL645882
S. meliloti 1021 Chromosome	GSE69880	December 12, 2017	NC_003047
S. meliloti 1021 pSymA	GSE69880	December 12, 2017	NC_003037
S. meliloti 1021 pSymB	GSE69880	December 12, 2017	NC_003078

Table S1: Strains and species used for each gene expression analysis. Gene Expression Omnibus accession numbers and date accessed are provided. NCBI genome accession numbers are listed for which genome was used to determine the gene position.

Origin and Terminus Locations

Bacteria	Origin of Replication	Terminus of Replication	Replicon Length (bp)
E. coli	3925744	1588773	4641652
B. subtilis	1	1942542	4215606
Streptomyces	3419363	1 & 8667507	8667507
S. meliloti Chromosome	1	1735626	3654135
S. meliloti pSymA	1350001	672888	1354226
S. meliloti pSymB	55090	896756	1683333

Table S2: Origin of replication and terminus of replication positions in replicons of representative strains of *E. coli*, *B. subtilis*, *Streptomyces*, and *S. meliloti*. The linear nature of *Streptomyces* chromosome gives it two termini, one at each end of the chromosome. The total base pair length for each bacterial replicon is additionally listed. Representative strain NCBI Accession Number can be found in Supplementary Table S1.

Correlation of Gene Expression Over Datasets

To assess uniform expression over bacteria with multiple data sets we looked at the mean normalized expression values. Multiple replicates from a data set were combined by finding the median normalized CPM expression value for each gene. This was done for any data sets that had multiple replicates. For each gene (x_i) the mean normalized expression value was calculated across all data sets ($\bar{x}_{ij}$). Then the

normalized median expression value for each data set was subtracted from the mean across all expression values ($|x_{ij} - \bar{x}_{ij}|$). The distribution of these $|x_{ij} - \bar{x}_{ij}|$ across all genes are found in Figures S1 and S2. All data sets are well mixed, implying that the expression levels are consistent across all data sets. Only *E. coli* and *B. subtilis* had multiple expression datasets available so they are the only ones that were analyzed. *Streptomyces* and all replicons of *S. meliloti* had only one data set each and therefore were not analyzed.

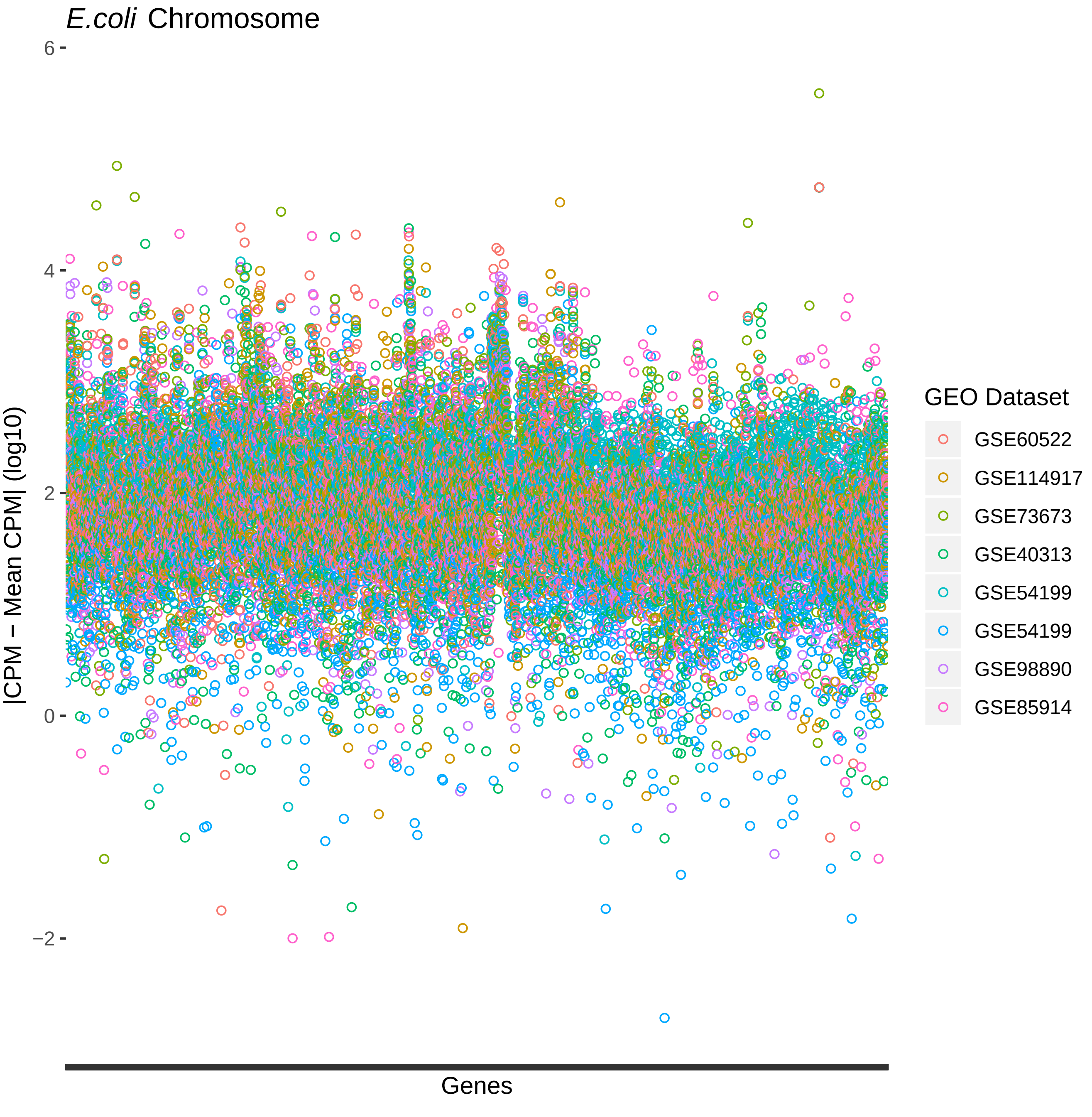

Figure S1: Dot plot distribution of the median expression value for each *E. coli* data set minus the mean expression value for that gene across all data sets. Each gene is shown on the x-axis and the log base 10 values are on the y-axis. The values are coloured by GEO data set.

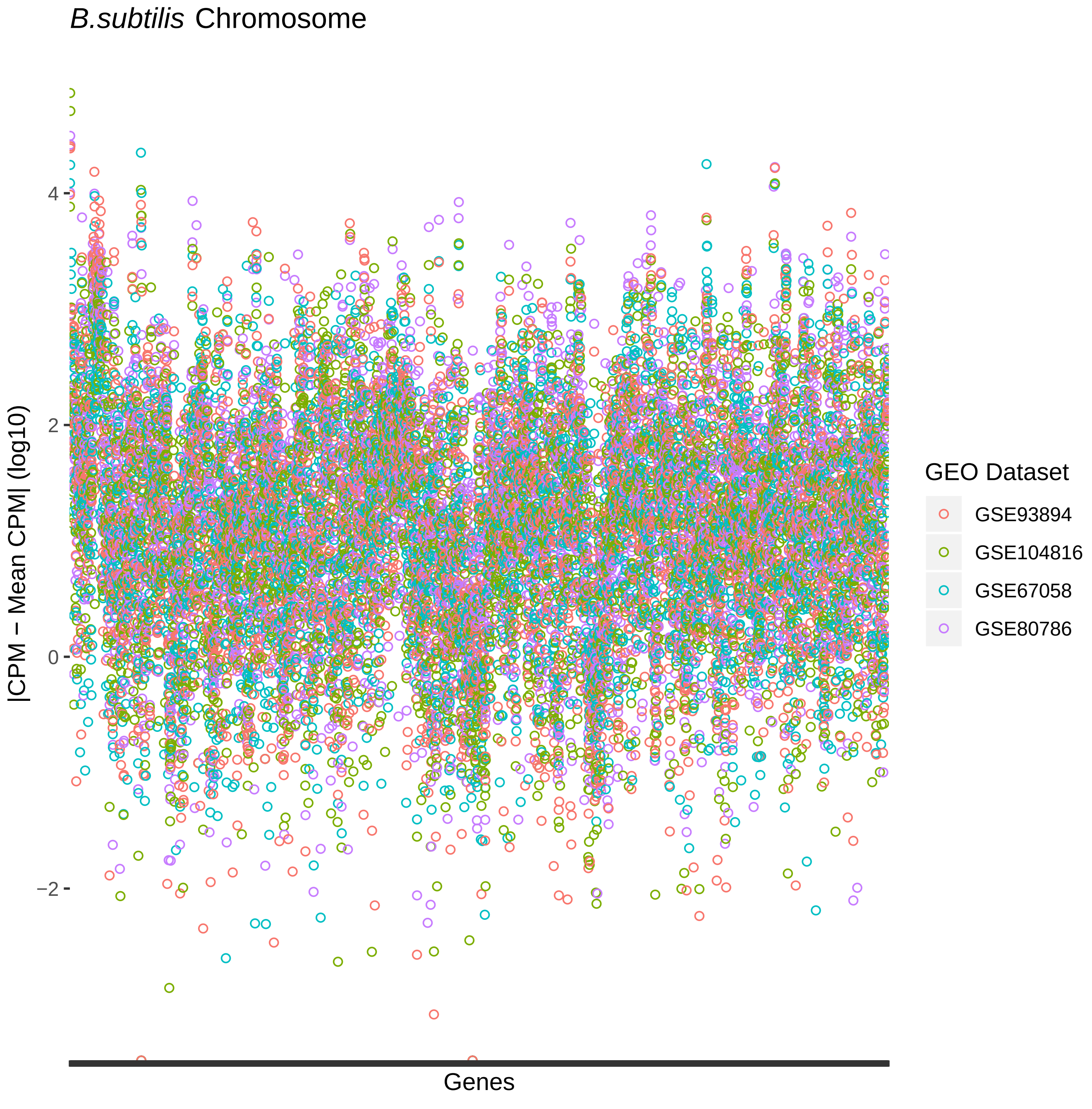

Figure S2: Dot plot distribution of the median expression value for each *B. subtilis* data set minus the mean expression value for that gene across all data sets. Each gene is shown on the x-axis and the log base 10 values are on the y-axis. The values are coloured by GEO data set.

Additional Linear Regression Tests

Multiple more detailed linear regressions are performed to determine if there is any correlation between gene expression per gene and distance from the origin of replication. A linear regression to determine how the median CPM expression values per gene changes with genomic position was performed. Additionally, a linear regression to determine how the median CPM expression value for each 10Kbp section of the genome changes with genomic position was performed. Finally, a linear regression to determine how the total added expression over each 10Kbp region of the genome changes with genomic position was performed. All linear regression results mirror the results from the linear regression on the median gene expression CPM value per gene. Most bacteria have a negative correlation, implying that gene expression tends to decrease with distance from the origin of replication.

We additionally performed a linear regression on a per gene basis. We found similar results as the linear regression of average expression values over 10Kbp regions: *E. coli* and *B. subtilis* had gene expression decrease with increasing distance from the origin of replication (Supplementary Table: S3). We were unable to detect a significant trend between gene expression and genomic position in the majority of the other bacterial replicons (Supplementary Table: S3). We performed a further linear regression tests on the median CPM gene expression value per 10Kbp region of the genome. This was calculated by determining the median CPM expression value across all genes in 10Kbp regions of the genome. We were able to detect similar results as the linear regression of average expression values over 10Kbp regions in *E. coli*, where median gene expression decreases with increasing distance from the origin of replication (Supplementary Table: S4). For all of the other bacterial replicons we were unable to determine a significant trend between median gene expression and genomic position (Supplementary Table: S4). Finally, we performed a linear regression test on the total additive CPM gene expression value per 10Kbp region of the genome. This was calculated by summing all gene CPM expression values across 10Kbp regions of the genome. We were able to detect similar results as the linear regression of average expression values over 10Kbp regions in most bacterial replicons where total gene expression decreases with increasing distance from the origin of replication (Supplementary Table: S5). For the two secondary replicons of *S. meliloti*, we were unable to detect a significant trend between total gene expression and genomic position (Supplementary Table: S5).

Bacteria and Replicon	Coefficient Estimate	Standard Error	P-value
E. coli Chromosome	-2.95×10^{-5}	1.29×10^{-5}	3.00×10^{-6}
B. subtilis Chromosome	-9.7×10^{-5}	2.0×10^{-5}	1.2×10^{-6}
Streptomyces Chromosome	-1.15×10^{-6}	8.12×10^{-8}	NS
S. meliloti Chromosome	2.85×10^{-5}	4.09×10^{-5}	NS
S. meliloti pSymA	1.39×10^{-3}	2.54×10^{-4}	5.48×10^{-8}
S. meliloti pSymB	1.47×10^{-4}	2.03×10^{-4}	NS

Table S3: Linear regression analysis of the median counts per million expression values per gene along the genome of the respective bacteria replicons. Linear regression was calculated after the origin of replication was moved to the beginning of the genome and all subsequent positions were scaled around the origin accounting for bidirectional replication. NS indicates Not Significant at $P \leq 0.05$. A grey row indicates a significant negative trend.

Bacteria and Replicon	Coefficient Estimate	Standard Error	P-value
E. coli Chromosome	-1.53×10^{-5}	3.91×10^{-6}	1.21×10^{-4}
B. subtilis Chromosome	-4.04×10^{-6}	2.82×10^{-6}	NS
Streptomyces Chromosome	-6.29×10^{-7}	3.27×10^{-8}	NS
S. meliloti Chromosome	2.19×10^{-6}	8.05×10^{-6}	NS
S. meliloti pSymA	-1.92×10^{-6}	1.03×10^{-4}	NS
S. meliloti pSymB	7.46×10^{-5}	7.03×10^{-5}	NS

Table S4: Linear regression analysis of the median counts per million expression data for 10Kbp segments of the genome of the respective bacteria replicons. Linear regression was calculated after the origin of replication was moved to the beginning of the genome and all subsequent positions were scaled around the origin accounting for bidirectional replication. Statistical outliers were removed from this linear regression calculation. NS indicates Not Significant at $P \leq 0.05$. A grey row indicates a significant negative trend.

Bacteria and Replicon	Coefficient Estimate	Standard Error	P-value
E. coli Chromosome	-3.41×10^{-4}	1.11×10^{-4}	2.47×10^{-3}
B. subtilis Chromosome	-5.63×10^{-4}	1.79×10^{-4}	1.87×10^{-3}
Streptomyces Chromosome	-1.37×10^{-6}	4.59×10^{-7}	2.88×10^{-3}
S. meliloti Chromosome	-6.97×10^{-4}	2.70×10^{-4}	1.08×10^{-2}
S. meliloti pSymA	9.04×10^{-3}	5.93×10^{-3}	NS
S. meliloti pSymB	-1.72×10^{-3}	2.30×10^{-3}	NS

Table S5: Linear regression analysis of total added expression and distance from the origin of replication. The total added expression values were calculated by summing the total counts per million expression value per 10Kbp section of the genome . Linear regression was calculated after the origin of replication was moved to the beginning of the genome and all subsequent positions were scaled around the origin accounting for bidirectional replication. NS indicates Not Significant at $P \leq 0.05$. A grey row indicates a significant negative trend.

Leading and Lagging Strand

A two-sample Wilcox test was computed to compare expression of genes on the leading strand and the lagging strand. We found that there was no significant difference between gene expression on the leading and lagging strand of any of the bacterial replicons.

Bacteria and Replicon	W	P-value	% of Genes on Leading Strand
E. coli Chromosome	1398352	0.9356	55.0
B. subtilis Chromosome	1678990.5	0.5736	73.8
Streptomyces Chromosome	7920836.5	1.75×10^{-5}	53.9
S. meliloti Chromosome	1462420	0.0124	55.6
S. meliloti pSymA	194005	0.3266	59.5
S. meliloti pSymB	297056.5	0.4736	55.9

Table S6: Two-sample Wilcox test results to determine if gene expression is significantly different between the leading and lagging strands of each bacterial replicon. The percentage of genes on the leading strand was also computed.

COG Analysis

A supplementary analysis of the spatial distribution of COG categories for each bacterial replicon was performed. For a full list of COG categories, please refer to Table S7.

This supplementary analysis shows that there appears to be no clear COG categories that are universally increasing or decreasing among the bacterial replicons in this analysis.

COG Data

Whole genomes of different strains and species of *E. coli*, *B. subtilis*, *Streptomyces* and *S. meliloti* were downloaded (Table S8). The analysis was performed on each replicon of multi-repliconic bacteria. For *S. meliloti* the analysis was performed on each of its replicons separately. The COG database information was downloaded on February 27, 2017 and spans the years 2003-2014. This data can be found on `GitHub` at (`https://github.com/dlato/Spatial_Patterns_of_Gene_Expression.git`) The only available data in the COG database for *Streptomyces* was for *Streptomyces bingchenggensis* and not *S. coelicolor*. We were therefore limited to using the annotation for *Streptomyces bingchenggensis*.

Using simple Python scripts,the COG protein ID and functional category was obtained for each known protein of each bacterial replicon in this analysis. This information was combined with the GenBank acession number and protein genome location to obtain the functional category of each protein and its midpoint location in the genome. The midpoint of each protein was calculated to be the singular point between the start and the end of the protein. This calculation was done to simplify the statistical calculations to verify the spatial trends of each COG category.

The origin and terminus of replication location, and bidirectional nature of bacterial replication were accounted for using the same methods as in the Gene Expression analysis. See "The Spatial Patters of Gene Expression in Bacterial Genomes" main paper for detailed methods.

COG Abbreviation	COG Category
A	RNA Processing and Modification
B	Chromatin Structure and Dynamics
C	Energy Production and Conversion
D	Cell Cycle Control and Mitosis
E	Amino Acid Transport and Metabolism
F	Nucleotide Transport and Metabolism
G	Carbohydrate Transport and Metabolism
H	Coenzyme Metabolis
I	Lipid Metabolism
J	Translation
K	Transcription
L	Replication and Repair
M	Cell Wall/Membrane/Envelope Biogenesis
N	Cell Motility
O	Post-translational Modification, Protein Turnover, Chaperone Functions
P	Inorganic Ion Transport and Metabolism
Q	Secondary Structure
T	Signal Transduction
U	Intracellular Trafficking and Secretion
V	Defence Mechanisms
W	Extracellular Structures
X	Mobilome: Prophages, Transposons
Y	Nuclear Structure
Z	Cytoskeleton
R	General Function Prediction Only
S	Function Unknown

Table S7: List of COG category letter abbreviation and full name of COG functional protein category.

COG Statistical Analysis

To determine if each COG category increased or decreased with increasing distance from the origin, a logistic regression was performed on each COG category for each replicon. Each of the proteins was considered present (1) or absent (0) in each COG category. Proteins that were classified under more than one COG category had a present (1) data point for each COG category. The binary nature of the COG data allowed for a simple logistic regression to be performed for each COG category using R. Logistic regression results are found in Table S9.

A visualization of the proportional distribution of the COG categories for each replicon can be seen in Figures:S3-S8.

COG Logistic Regression Results

Bacteria Strain	Accession Number	Date Accessed
E. coli K12	U00096	September 26, 2016
Bacillus subtilis 168	NC_000964	November 10, 2016
Streptomyces bingchenggensis BCW1	CP002047	June 7, 2017
S. meliloti 1021	NC_003047	June 3, 2014

Table S8: List of bacteria genomes used for the COG category information. This includes the accession number and date accessed.

COG Category	*E. coli* Chromosome	*B. subtilis* Chromosome	*Streptomyces* Chromosome	*S. meliloti* Chromosome	*S. meliloti* pSymA	*S. meliloti* pSymB
RNA Processing and Modification	NS	NS	NS	NS	NS	NS
Chromatin Structure and Dynamics	NS	NS	NS	NS	NS	NS
Energy Production and Conversion	2.40×10^{-7}	4.10×10^{-7}	-2.94×10^{-7}	NS	NS	NS
Cell Cycle Control and Mitosis	NS	NS	NS	NS	NS	NS
Amino Acid Transport and Metabolism	4.53×10^{-7}	NS	-1.97×10^{-7}	2.66×10^{-7}	-2.08×10^{-6}	-9.45×10^{-7}
Nucleotide Transport and Metabolism	NS	-7.49×10^{-7}	-1.86×10^{-7}	-6.68×10^{-7}	-1.15×10^{-6}	NS
Carbohydrate Transport and Metabolism	NS	NS	NS	-2.53×10^{-7}	9.78×10^{-7}	2.05×10^{-6}
Coenzyme Metabolis	NS	-4.07×10^{-7}	-1.11×10^{-7}	-1.20×10^{-6}	-9.83×10^{-7}	-1.45×10^{-6}
Lipid Metabolism	4.51×10^{-7}	3.74×10^{-7}	-2.01×10^{-7}	NS	2.01×10^{-6}	1.84×10^{-6}
Translation	NS	-7.13×10^{-7}	-1.36×10^{-7}	1.23×10^{-6}	-1.51×10^{-6}	-1.15×10^{-6}
Transcription	2.22×10^{-7}	7.62×10^{-7}	NS	NS	NS	-4.17×10^{-6}
Replication and Repair	2.95×10^{-7}	NS	-1.17×10^{-7}	NS	1.42×10^{-6}	NS
Cell Wall/Membrane/Envelope Biogenesis	NS	5.18×10^{-7}	-8.05×10^{-8}	4.59×10^{-7}	1.63×10^{-6}	5.41×10^{-6}
Cell Motility	-7.74×10^{-7}	1.01×10^{-6}	-2.04×10^{-7}	NS	NS	NS
Post-translational Modification, Protein Turnover, Chaperone Functions	3.37×10^{-7}	3.51×10^{-7}	-7.75×10^{-8}	3.47×10^{-7}	NS	1.08×10^{-6}
Inorganic Ion Transport and Metabolism	NS	NS	-1.68×10^{-7}	5.36×10^{-7}	NS	-2.05×10^{-6}
Secondary Structure	NS	NS	NS	NS	4.28×10^{-6}	3.81×10^{-6}
Signal Transduction	NS	NS	1.52×10^{-7}	1.85×10^{-6}	NS	NS
Intracellular Trafficking and Secretion	NS	NS	NS	8.62×10^{-7}	NS	NS
Defence Mechanisms	3.75×10^{-7}	7.15×10^{-7}	-1.21×10^{-7}	4.24×10^{-7}	NS	NS
Extracellular Structures	-3.23×10^{-6}	NS	9.06×10^{-7}	NS	NS	NS
Mobilome: Prophages, Transposons	-1.09×10^{-6}	-1.81×10^{-6}	4.32×10^{-7}	1.67×10^{-6}	NS	-3.35×10^{-6}
Nuclear Structure	NS	NS	NS	NS	NS	NS
Cytoskeleton	NS	NS	NS	NS	NS	NS
General Function Prediction Only	2.61×10^{-7}	3.20×10^{-7}	-6.91×10^{-8}	9.49×10^{-7}	NS	NS
Function Unknown	-1.43×10^{-6}	-1.19×10^{-6}	4.62×10^{-7}	-7.44×10^{-7}	2.53×10^{-5}	4.14×10^{-5}

Table S9: Logistic regression coefficients for each bacterial replicon analysis showing the change in each COG category with increasing distance from the origin of replication. Only statistically significant ($p < 0.05$) coefficient estimates are shown in the table. Any values of NS did not have a statistically significant p-value. Grey cells indicate logistic regression coefficients that were negative.

High Gene Expression Distribution

Bacteria and Replicon	Bidirectional Genomic Position (bp)	Protein/Gene Examples
E. coli Chromosome	0 - 10000	DNA replication and repair
		ATP-proton motive force
		ATP biosynthesis
		transport
	470000 - 480000	DNA replication and repair
		tRNA synthesis
		Ribosomal proteins
		Putative transport
	610000 - 620000	Ribosomal protein
		Translation modification
		tRNA modification
		RNA synthesis
	840000 - 850000	Energy metabolism
	1170000 - 1180000	Cell division
		Protein synthesis modification
B. subtilis Chromosome	0 - 10000	tRNA modification
		Ribosomal proteins
		DNA gyrase
		rRNA small subunit methylation
	130000 - 150000	Ribosomal proteins
		Elongation factor
	730000 - 740000	tRNA subunit
		Transcription regulation
		Glycolysis
Streptomyces Chromosome	1700000 - 1720000	Ribosomal proteins
		RNA Polymerase alpha chain
	1200000 - 1210000	Possible ATP-binding proteins
		Putative oxidoreductase
		Integral membrane proteins
	-2900000 - -2890000	Putative peptide synthetase
S. meliloti Chromosome	630000 - 640000	Cell processes
		Structural Elements
	1480000 - 1490000	Ribosomal proteins
		Structural elements
		Transmembrane proteins
S. meliloti pSymA	0 - 20000	Cell processes
		Hypothetical proteins
	660000 - 680000	Small molecule metabolism
		Not classified regulator
		Glimmer prediction
		Hypothetical protein
S. meliloti pSymB	210000 - 220000	Unknown proteins
		Cell processes
		Hypothetical proteins
	290000 - 300000	Cell Division
		Small molecule metabolism
		Cell processes

	790000 - 820000	Small molecule metabolism<br>Cell processes

Table S10: Table of high median CPM (Counts per Million) gene expression over 10Kbp genomic regions for each bacterial replicon and the associated proteins/gene functions found in that region. The genomic position begins at the origin of replication and continues in both directions until the terminus of replication (bidirectional replication).

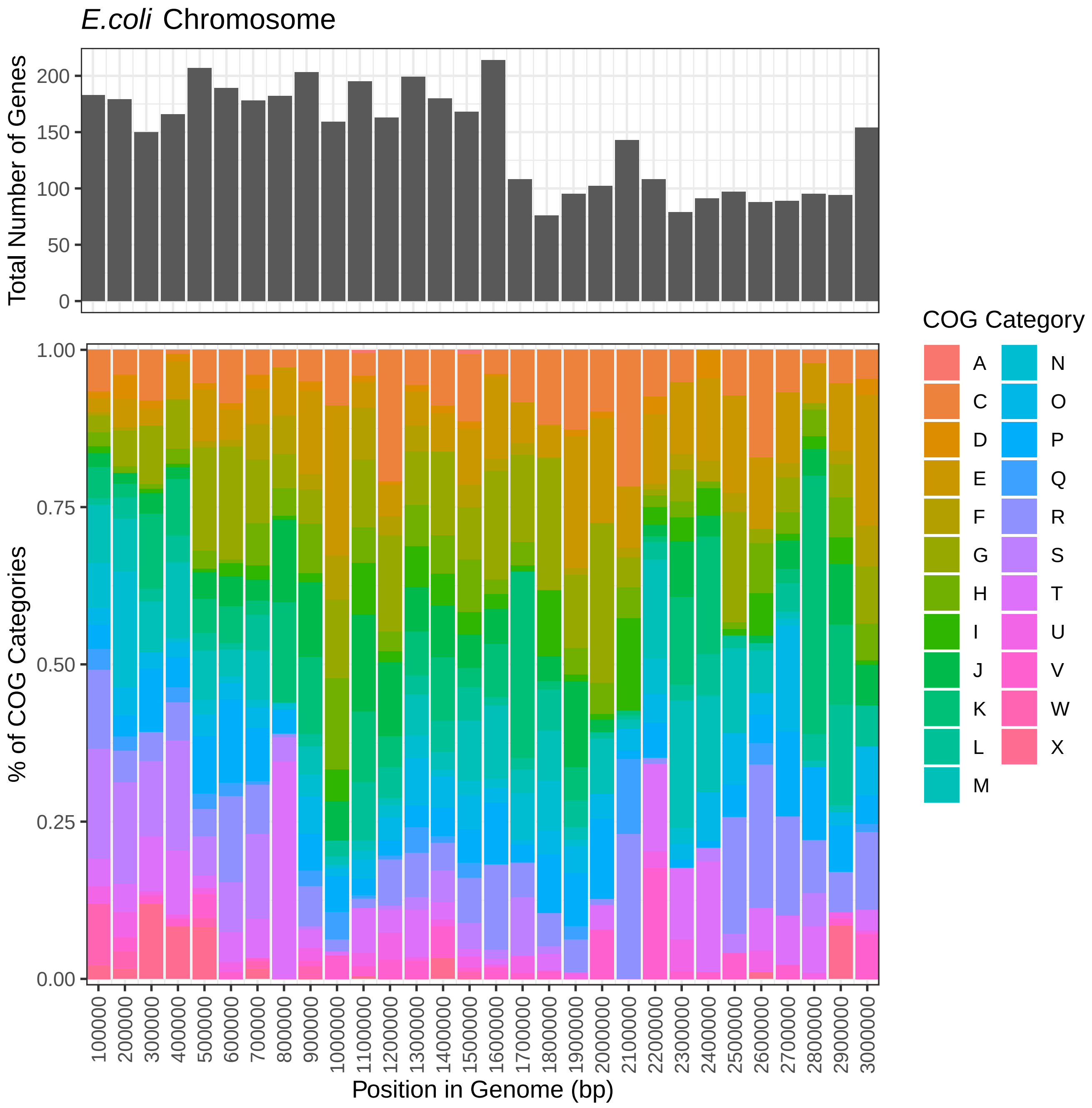

Figure S3: Graphical representation of COG categories across the chromosome of *E. coli*. Bidirectional distance from the origin of replication is along the x-axis. Each bar represents a 100Kbp segment of the genome. The grey graph represents the total number of genes in each 100Kbp section of the genome. The colourful graph represents the percentage of COG categories in each 100Kbp section of the genome. The full name for each COG category can be found in Table S7.

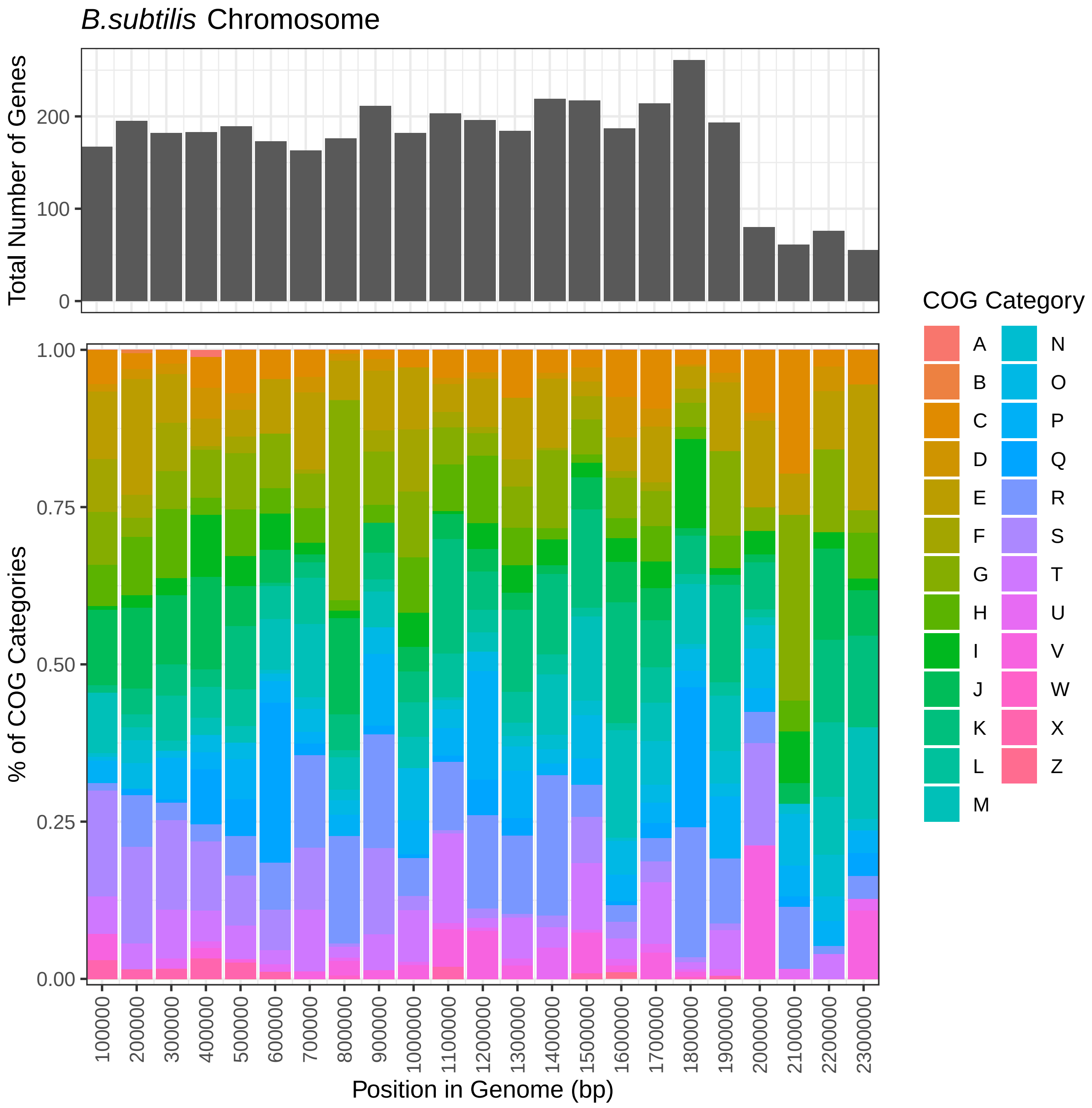

Figure S4: Histogram of COG categories across the chromosome of *B. subtilis*. Bidirectional distance from the origin of replication is along the x-axis. Each bar represents a 100Kbp segment of the genome. The grey graph represents the total number of genes in each 100Kbp section of the genome. The colourful graph represents the percentage of COG categories in each 100Kbp section of the genome. The full name for each COG category can be found in Table S7.

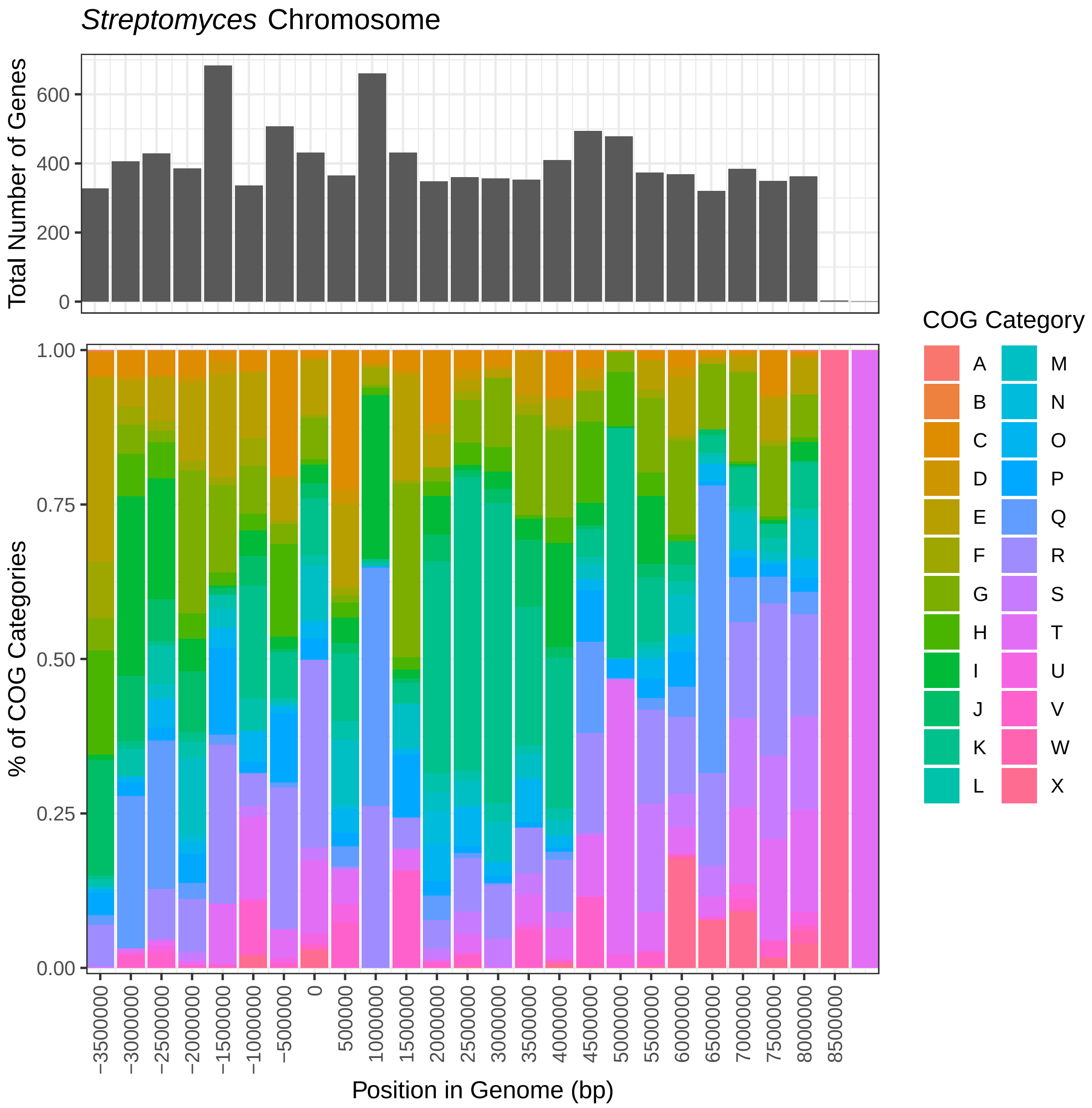

Figure S5: Histogram of COG categories across the chromosome of *Streptomyces*. Distance from the origin of replication is along the x-axis with the origin of replication denoted by position 0. The genome located on the shorter chromosome arm (to the left of the origin) has been given negative values, while the genome on the longer chromosome arm (to the right of the origin) has been given positive values. Each bar represents a 500Kbp segment of the genome. The grey graph represents the total number of genes in each 500Kbp section of the genome. The colourful graph represents the percentage of COG categories in each 500Kbp section of the genome. The two bars on the far right side of the graph have only one COG category present due to under representation of annotated genes in those sections of the genome. The full name for each COG category can be found in Table S7.

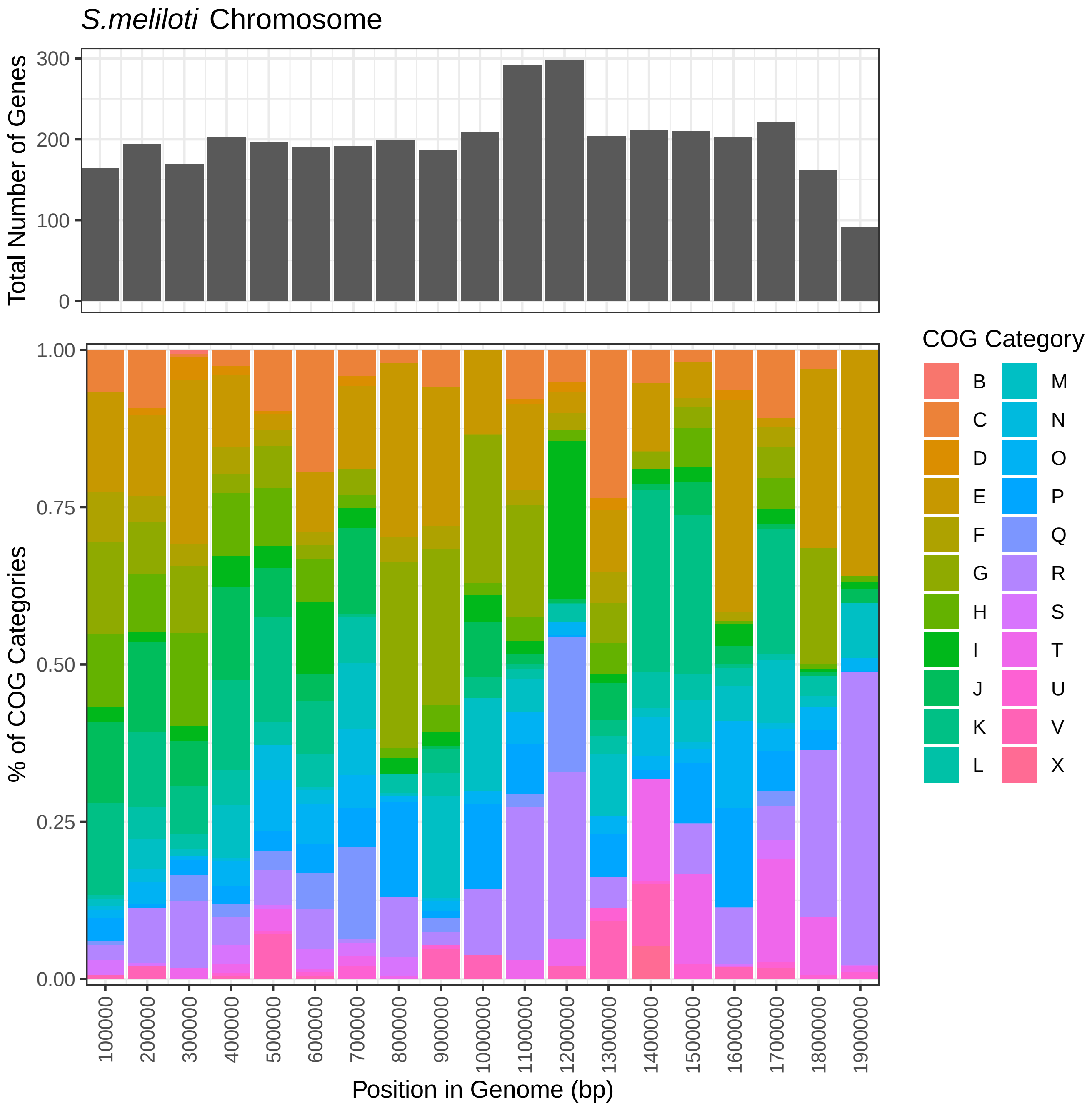

Figure S6: Histogram of COG categories across the chromosome of *S. meliloti*. Bidirectional distance from the origin of replication is along the x-axis. Each bar represents a 100Kbp segment of the genome. The grey graph represents the total number of genes in each 100Kbp section of the genome. The colourful graph represents the percentage of COG categories in each 100Kbp section of the genome. The full name for each COG category can be found in Table S7.

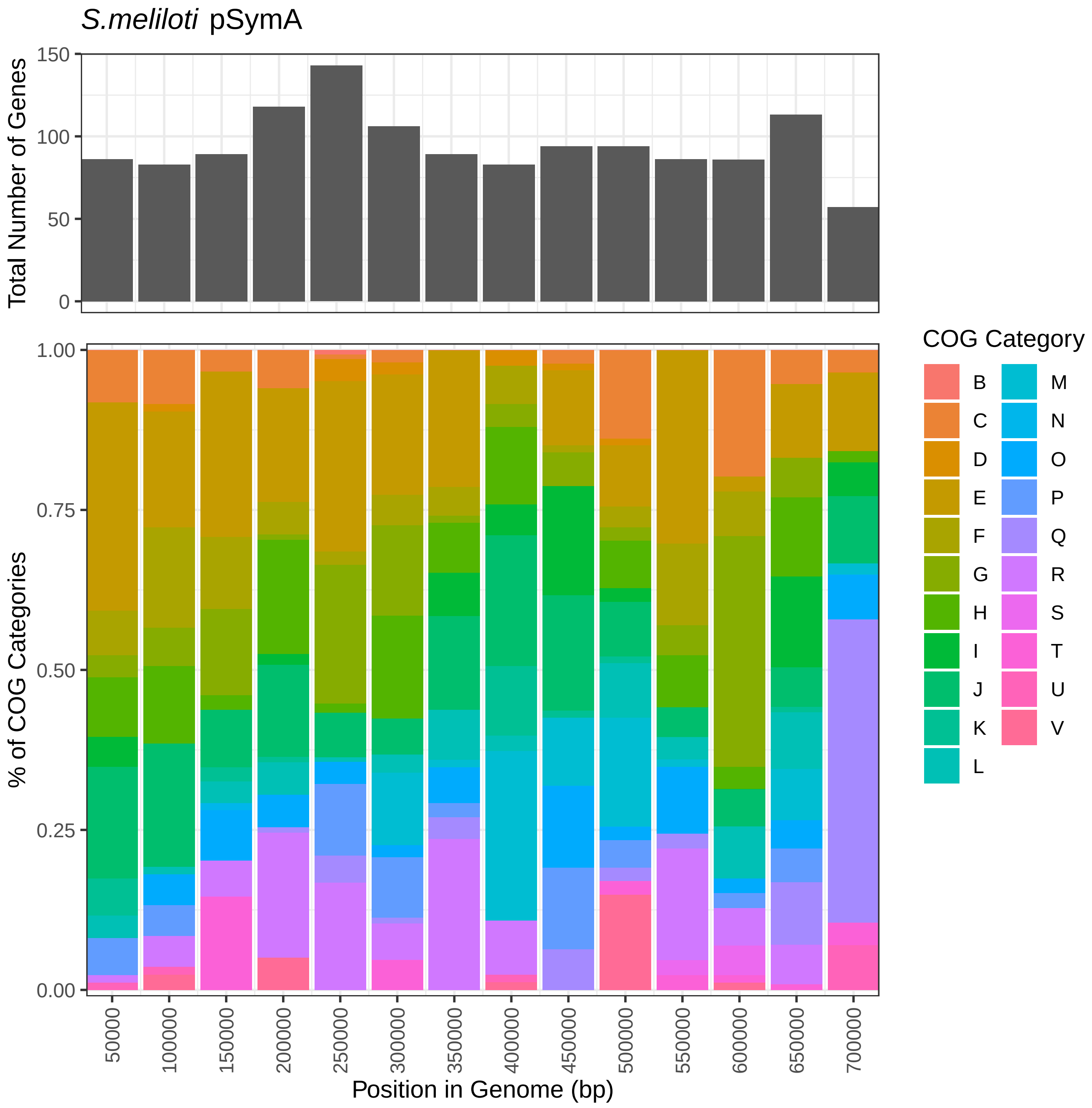

Figure S7: Histogram of COG categories across pSymA of *S. meliloti*. Bidirectional distance from the origin of replication is along the x-axis. Each bar represents a 50Kbp segment of the genome. The grey graph represents the total number of genes in each 50Kbp section of the genome. The colourful graph represents the percentage of COG categories in each 50Kbp section of the genome. The full name for each COG category can be found in Table S7.

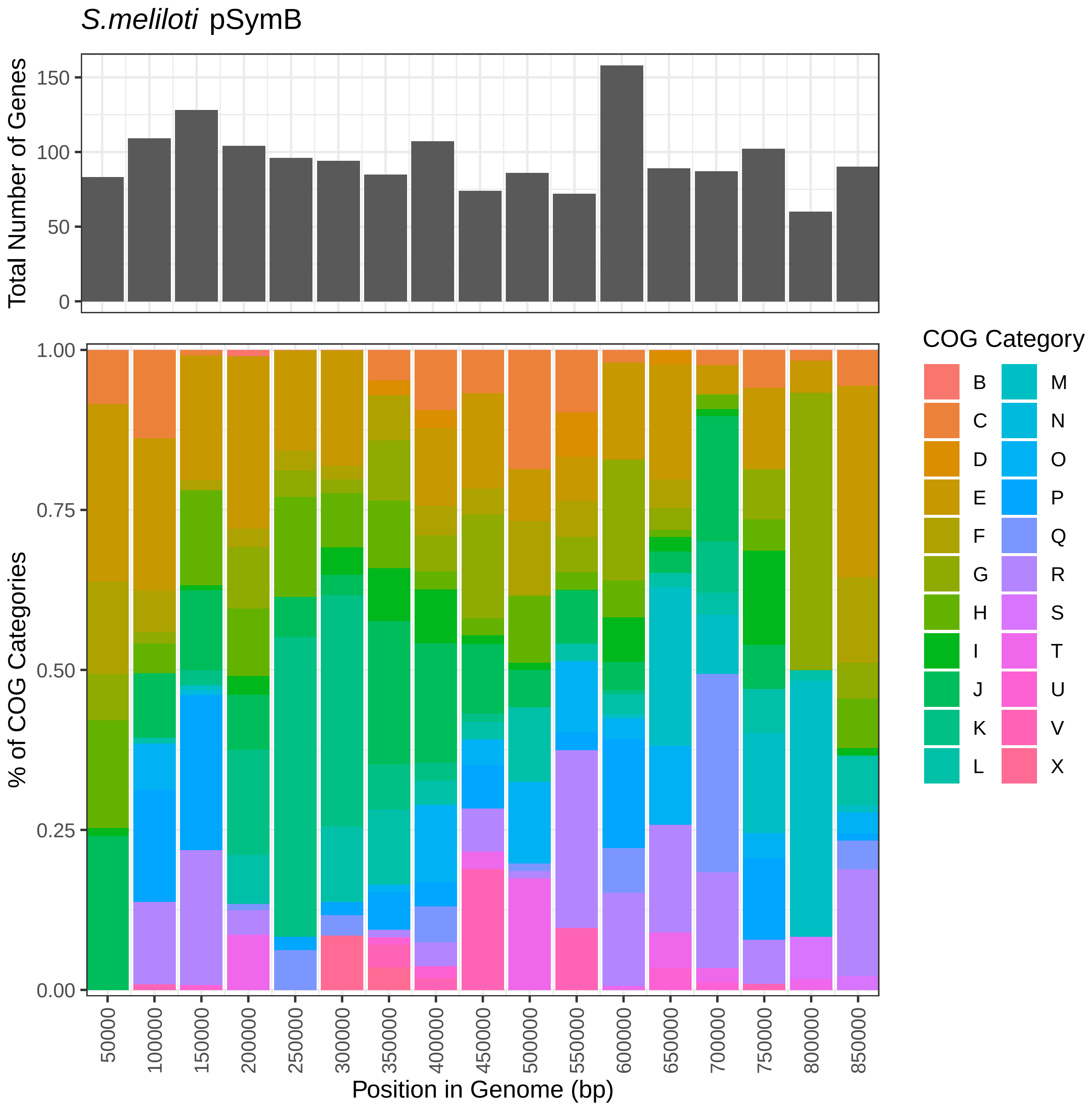

Figure S8: Histogram of COG categories across pSymA of *S. meliloti*. Bidirectional distance from the origin of replication is along the x-axis. Each bar represents a 50Kbp segment of the genome. The grey graph represents the total number of genes in each 50Kbp section of the genome. The colourful graph represents the percentage of COG categories in each 50Kbp section of the genome. The full name for each COG category can be found in Table S7.
